# Geometry-Dependent Transverse Shear Stiffness of Corrugated Board

**DOI:** 10.3390/ma19204280

**Published:** 2026-10-10

**Authors:** Tomasz Garbowski, Anna Knitter-Piątkowska, Jolanta Pozorska, Tomasz Stręk

**Affiliations:** 1 University Center for Ecomaterials, Poznan University of Life Sciences, Wojska Polskiego 50, 60-627 Poznań, Poland; 2 Institute of Structural Analysis, Poznan University of Technology, Piotrowo 5, 61-131 Poznań, Poland; 3 Institute of Mathematics, Poznan University of Technology, Piotrowo 3a, 61-131 Poznań, Poland; jolanta.pozorska@put.poznan.pl; 4 Institute of Applied Mechanics, Poznan University of Technology, Jana Pawła II 24, 61-131 Poznań, Poland; tomasz.strek@put.poznan.pl

**Keywords:** corrugated board, flute geometry, transverse shear, stiffness, sensitivity analysis, finite element method

## Abstract

This study examines how flute shape and geometric proportions govern the effective transverse shear stiffness of corrugated-board cores. Six profiles are compared at common nominal dimensions and material properties using a strain-energy estimate, the Timoshenko frame model for shear in the corrugation plane, and a membrane-path model for shear along the flute axis. Finite element solutions and mesh refinement provide numerical verification. For the reference configuration, the predicted core shear moduli span 1.52–106.36 MPa in the corrugation plane and 38.20–60.00 MPa along the flute axis. The directional rankings reflect different contributions from axial deformation, bending, and shear along the free flute path. The triangular profile combines the highest frame shear modulus with the lowest material consumption, whereas the rectangular profile gives the highest membrane shear modulus under full plateau bonding. Parametric analyses show that flute height, pitch, and wall thickness affect the two directions differently, with distinct trends for shear modulus and rigidity. The framework provides a reproducible basis for comparing flute geometries within small-strain linear elasticity under rigid face constraints and ideal bonding; predictive accuracy for manufactured board requires experimental validation.

## 1. Introduction

Corrugated board is a lightweight periodic sandwich material whose macroscopic mechanical response results from the interaction between the orthotropic paper constituents and the geometry of the corrugated medium. Because direct representation of the flute architecture is computationally expensive at the scale of complete packages, analytical homogenization and equivalent-property finite element formulations have become important tools for describing its structural behavior [1,2,3,4]. Among the effective constitutive parameters, transverse shear stiffness is particularly important in bending-dominated problems, where shear deformation may contribute significantly to global deflections and influence the accuracy of equivalent plate or shell models [5,6,7,8,9,10].

The transverse shear response is strongly geometry-dependent because the corrugated medium acts as a periodic load-transfer path whose local inclination, length, and connectivity are controlled by the flute geometry. Experimental and numerical studies have addressed the measurement and modeling of transverse shear behavior [5,6,7,8,9], while other investigations have examined the influence of flute configuration on flat-crush resistance, structural performance, or the mechanics of more general corrugated-core systems [11,12,13,14,15,16]. These studies have demonstrated that changes in waveform geometry may alter the mechanical response. However, they were performed for different loading conditions, materials, geometric constraints, and modeling assumptions, which makes it difficult to determine whether the observed differences originate from the exact waveform shape itself or simply from changes in global geometric proportions.

A further difficulty arises from the geometric representation adopted in analytical and numerical models. Classical homogenization approaches frequently rely on idealized periodic descriptions of the corrugated medium [1,2,3,4], whereas more recent finite element models have focused on efficient representation and validation of board- or package-level response [17,18]. Other studies have considered specific mechanisms such as shear instability of the corrugated core [19]. Consequently, the available literature contains a broad spectrum of mechanical models but relatively little systematic information on how the predicted effective transverse shear stiffness changes when several flute families are compared under identical dimensions, material parameters, and kinematic assumptions. This distinction becomes especially relevant for profiles containing flat regions, steep webs, rounded corners, or slope discontinuities, for which formulations involving curvature may behave differently from those developed for smooth sinusoidal geometries.

Two earlier studies involving the present authors address related but distinct questions. Ref. [9] examines the role and identification of transverse shear moduli in corrugated materials. Ref. [20] investigates nonlinear transverse shear response and failure for six conceptual flute geometries. The present manuscript concentrates on small-strain effective stiffness and the assumptions used to compare analytical and beam finite element descriptions. This distinction concerns the response quantity and modeling scope; it does not imply that geometry-dependent shear response has not previously been studied.

Recent homogenization studies help delineate the scope of such a comparison. Liang and Wang [21] include transverse normal strain in a corrugated-cardboard plate element, addressing a thickness-deformation mode excluded from a constant-thickness description. Graczyk et al. [22] examine geometry and material-contrast effects on shear correction factors across single- and double-wall configurations. These studies show why conclusions depend on the selected deformation modes and parameter domain; neither supplies experimental validation of the six-profile beam comparison considered here.

Geometry-induced coupling has also been investigated in other architected materials. Giannopoulos and Mostralis [23] consider compression-induced twist in three-dimensional printed lattice metamaterials. This is relevant as a broader example of geometry-dependent response, but its material system and loading mode differ from transverse shear of paperboard, so its results are not used as a quantitative benchmark here.

At the model level, the literature progresses from analytical structural-core shear descriptions [24] to energy-equivalent plate homogenization of detailed finite element cells [25,26] and then to models incorporating initial imperfections [27] or identifying converted-board properties through inverse analysis [28]. These approaches address effective-property extraction or manufacturing effects; the present study uses prescribed ideal geometries to resolve how profile-dependent axial, bending, and shear contributions affect the two transverse directions under common conditions.

This distinction defines the specific gap addressed in the present study. For a corrugated board with fixed constituent properties and nominal dimensions, it remains unclear how much of the effective transverse shear stiffness can be attributed to the exact profile family and how much is governed by more fundamental geometric quantities such as flute height, pitch, wall thickness, and the unfolded material path. Answering this question is relevant not only for homogenization but also for practical flute design because modifying a waveform may increase manufacturing complexity or material consumption without producing a proportional increase in structural stiffness.

The present study compares six idealized flute families under common nominal dimensions and material inputs. The affine axial estimate is contrasted with an equilibrium frame solution that admits internal relaxation. A force-flexibility formulation integrated along the exact centerline is checked against a separately assembled beam finite element model. The yz direction is treated by a distinct scalar membrane-path model. These distinctions are necessary because changing coordinates alone does not define another mechanical model, and a planar xz frame cannot supply a yz shear solution.

This study provides a reproducible comparison with explicit profile definitions, contact constraints, dimensional stiffnesses, and mesh-refinement results. It examines how waveform and geometric proportions affect different shear mechanisms under a fixed contact convention. The trapezoidal flat fraction and corner radius are specified inputs, and numerical verification is separated from assessment of physical model accuracy. Its specific contribution is the joint comparison of six explicitly defined profiles, directional mechanisms, and material-path efficiency under one reproducible set of geometric and contact assumptions.

## 2. Materials and Methods

The effective response is evaluated with three distinct mechanical descriptions: prescribed affine axial strain, a relaxed Timoshenko frame for xz shear, and membrane shear along the corrugated path for yz shear. Force-flexibility integration and finite element (FE) assembly are two solution routes for the frame model, not two different constitutive theories.

All principal reported moduli refer to the corrugated core between rigid translating contact levels. Finite-thickness liners and adhesive layers are not discretized. Section 2.6 gives an optional board-level compliance relation but does not supply numerical whole-board properties in the absence of liner transverse shear data.

A finite element model was used as a numerical comparison within the same beam-type mechanical idealization. Agreement with the analytical formulations can indicate consistency between implementations but cannot independently validate their shared kinematic assumptions or establish accuracy for manufactured corrugated board.

In all cases, the objective was to isolate the role of flute geometry in the effective transverse shear response while keeping the material parameters identical across the compared profiles.

### 2.1. Geometry and Notation

**Figure 1:**
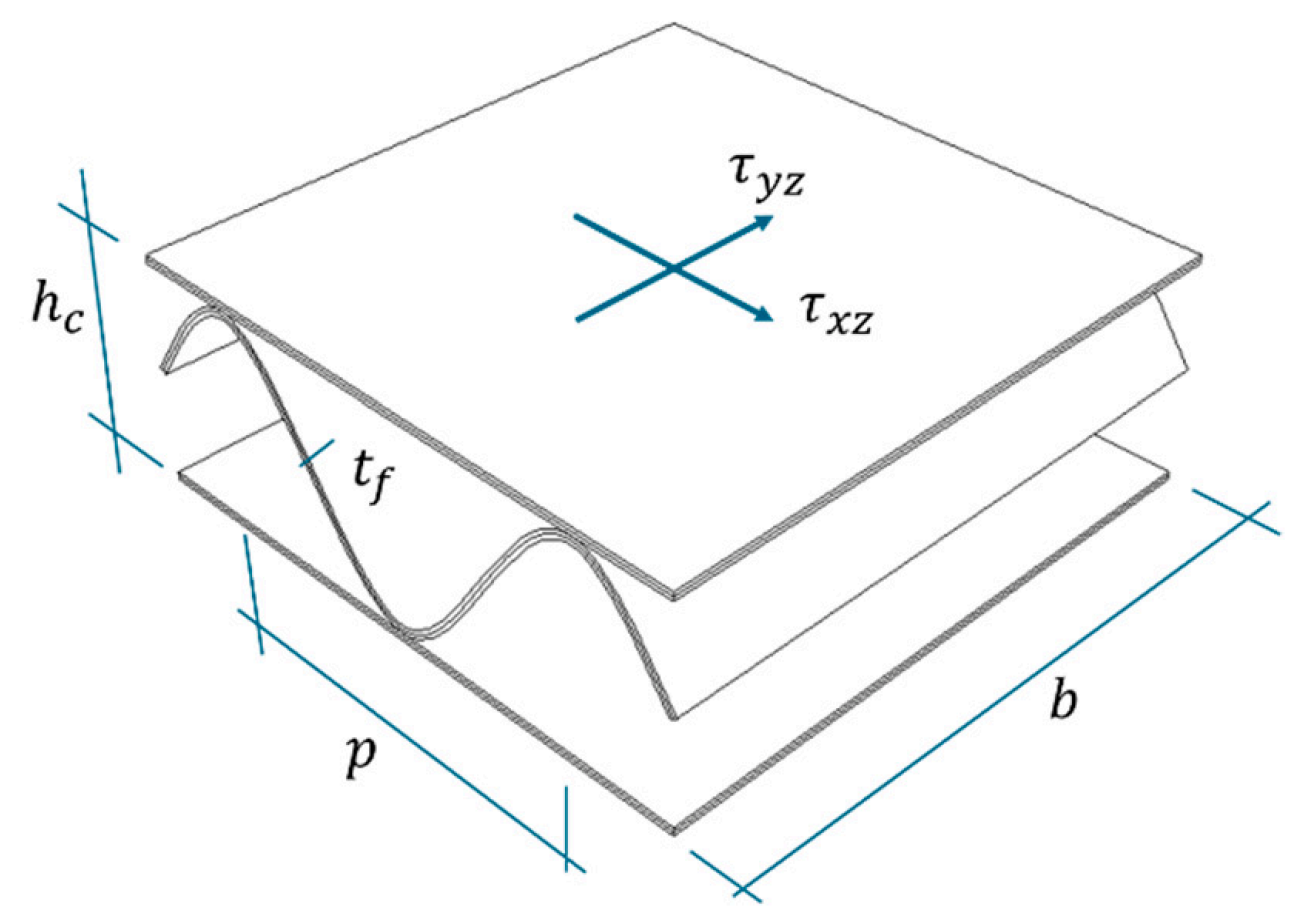
Geometry of the corrugated board unit cell and notation used in the study: flute pitch *p*, core height *h_c_*, flute-wall thickness *t_f_*, liner thickness *t_l_*, and machine (MD) and cross (CD) directions.

A periodic corrugated medium between two idealized rigid face levels is considered (Figure 1). The nominal liner thickness is retained as geometric context, but the numerical unit cell includes the fluted medium only. Rigid face constraints represent ideal contact and suppress face deformation; this is a stated modeling assumption rather than a representation of the full liner–flute assembly.

For profiles that admit a single-valued graph, the flute centerline in the x–z plane is written as

*z* = *z*(*x*),     *x* ∈ [0, *p*] (1)

where *x* denotes the in-plane coordinate over one flute pitch, *p* is the corrugation pitch (period), and *z* represents the local position of the flute centerline measured relative to the reference mid-plane of the board.

The total height of the corrugated core is denoted by *h_c_*, the flute-wall thickness by *t_f_*, and the thickness of each liner by *t_l_*. Unless stated otherwise, both liners were assumed identical. The total board thickness is denoted by *h*.

The x direction spans one corrugation pitch and is perpendicular to the flute axis; y is parallel to the flute axis, and z is the thickness direction. For conventional corrugated-board production, x corresponds to MD and y to CD. Constituent paper moduli must be assigned consistently with these axes. Thus the xz and yz responses represent physically distinct shear modes and need not have equal moduli or equal normalized shape dependence.

The xz response involves deformation in the corrugation plane, whereas the yz response involves shear along the flute axis. These directional definitions are used throughout the study.

The unfolded length of one flute period is defined as

(2) $$L=\int_{0}^{p}\sqrt{1+{\left(z^{\prime }\left(x\right)\right)}^{2}}\;\mathrm{dx}$$

which is the centerline length obtained by flattening one period without stretching the paper. For piecewise-smooth profiles, lengths are summed over the individual segments. An exactly rectangular path with vertical webs is not a single-valued function z(x) and must instead be described parametrically by arc length.

The ratio of the unfolded flute length to the pitch,

(3) $$T=\frac{L}{p}$$

is the dimensionless take-up factor. At fixed paper thickness, density, and out-of-plane width, T is also the ratio of the corrugated-medium mass to that of a flat strip covering the same projected pitch. A flat strip has T = 1, whereas a non-flat flute has T > 1. This indicator concerns the corrugated medium alone; it excludes liners, adhesive, and manufacturing waste. A larger T therefore means greater medium consumption but does not by itself imply greater shear stiffness.

All profiles use the same pitch, core-height convention, paper thickness, and assigned constitutive values. The contact set follows the geometry: isolated extrema for smooth or triangular profiles and entire extreme-height horizontal segments for profiles with flat plateaus. Consequently, waveform changes also change the bonded contact length under this explicit convention.

### 2.2. Basic Analytical Model Based on Strain-Energy Equivalence

A first estimate of the effective transverse shear stiffness was obtained using a strain-energy equivalence approach applied to one periodic flute segment. The corrugated medium was represented by its centerline profile *z*(*x*), defined over one pitch *p*.

For the basic kinematic estimate, a macroscopic engineering shear strain Γ is represented by the horizontal displacement *u_x_* = Γz, with *u_z_* = 0. Evaluated on the flute centerline, this gives

*u_x_*(*x*) = *Γz*(*x*) (4)

**Figure 2:**
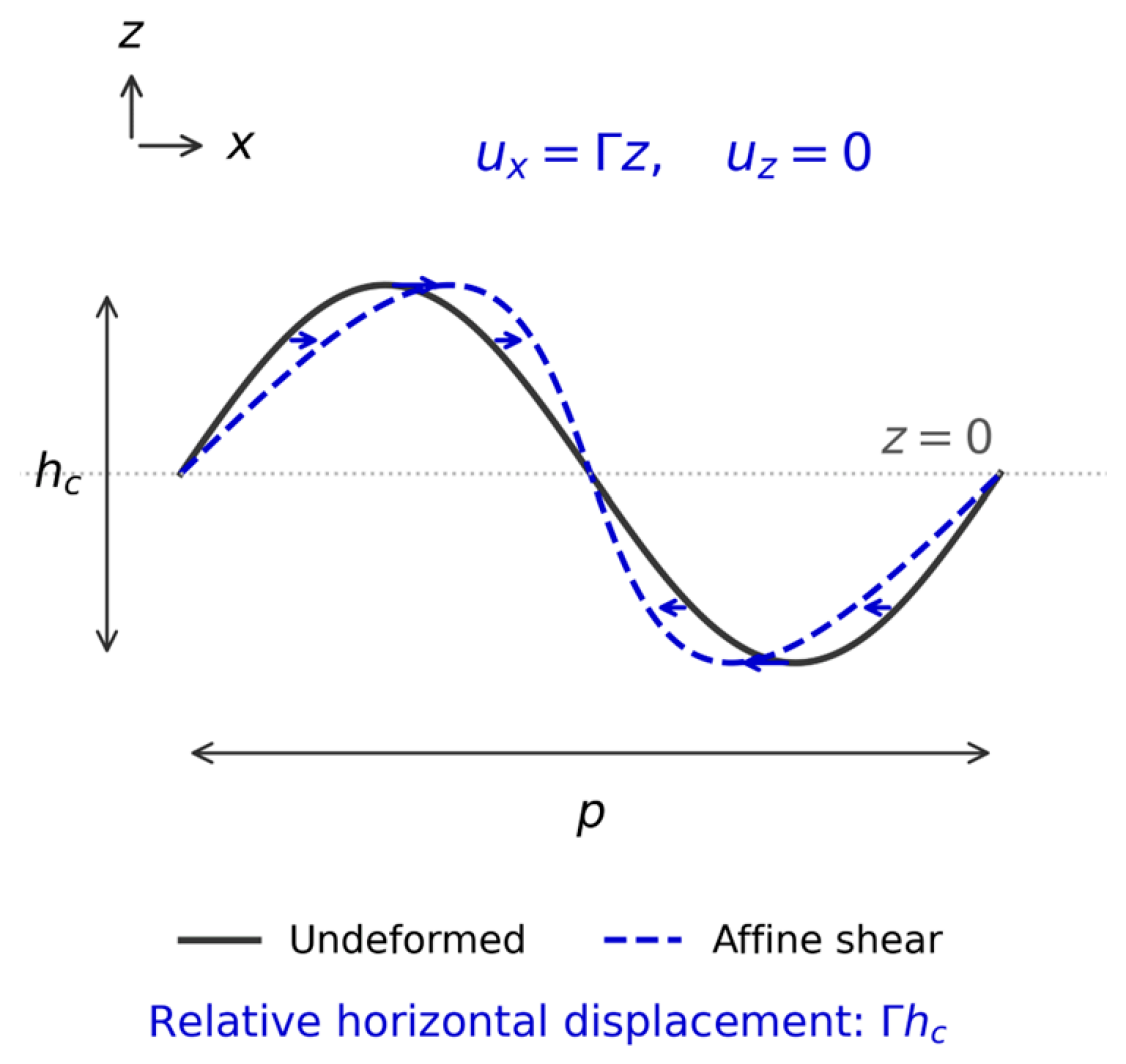
Affine shear trial field used in the basic energy estimate. Horizontal displacements satisfy *u_x_* = Γz and *u_z_* = 0; the relative displacement between crest and trough is Γ*h_c_*. The deformation is exaggerated for clarity and is not an FE displacement solution.

This field produces *γ_xz_* = ∂*u_x_*/∂z + ∂*u_z_*/∂x = Γ and a relative horizontal displacement Γ*h_c_* between the crest and trough levels. The displacement is parallel to x, not vertical. Figure 2 illustrates this affine shear field. It is a prescribed trial kinematics, not an assertion that the actual microscopic displacement remains affine after equilibrium relaxation.

For the unit tangent $t\;=\;\left(1,\;z^{\prime }\right)/\sqrt{1\;+{\left(z^{\prime }\right)}^{2}}$, the local axial strain is $\varepsilon_{s}=t\cdot \varepsilon \cdot t\;=\Gamma z^{\prime }/\left(1\;+{\left(z^{\prime }\right)}^{2}\right)$. Thus the macroscopic shear projects onto stretching of inclined wall segments. The axial strain energy per unit out-of-plane width is

(5) $$U_{\mathrm{axial}}=\frac{1}{2}\int_{0}^{L}\mathrm{EA}\;\varepsilon_{s}^{2}\;\mathrm{ds}$$

where *E* is the paper modulus along the flute path, *A* = *t_f_* per unit width, and *L* is the unfolded length. Energy is expressed per unit out-of-plane width throughout this derivation.

The equivalent homogenized unit cell is characterized by the transverse shear stiffness *K_xz_*, whose macroscopic energy under the same strain is

(6) $$U_{\mathrm{eq}}=\frac{1}{2}K_{\mathrm{xz}}\Gamma^{2}p$$

By imposing energy equivalence, the resulting effective stiffness becomes

(7) $$K_{\mathrm{xz}}=\frac{Et_{f}}{p}\int_{0}^{p}\frac{{\left(z^{\prime }(x)\right)}^{2}}{{\left(1+{\left(z^{\prime }\left(x\right)\right)}^{2}\right)}^{3/2}}\mathrm{dx}$$

and the corresponding effective shear modulus is

(8) $$G_{\mathrm{xz}}=\frac{K_{\mathrm{xz}}}{h_{c}}$$

This expression measures only the axial energy of the prescribed affine field. It is not the relaxed frame stiffness and is not an upper or lower bound on a model with a different set of energy terms. In particular, a rectangular path gives zero axial contribution because each segment is horizontal or vertical; its nonzero frame response arises from bending and local shear.

### 2.3. Relaxed Frame Model Including Bending and Local Shear

The equilibrium model treats each unbonded web as an extensible Timoshenko member of area A = *t_f_* and second moment I = *t_f_*^3^/12 per unit width. E = *E_MD_* = 4000 MPa acts along the material path, while kG = (5/6)800 MPa supplies the local transverse shear coefficient. These are independent assigned moduli; no isotropic E–G–ν relation is imposed.

The total elastic energy contains axial, incremental bending, and local shear terms:

*U* = *U*_axial_ + *U*_bend_ + *U*_shear_ (9)

where

(10) $$U_{\mathrm{bend}}=\frac{1}{2}\int_{0}^{L}\mathrm{EI}\;\kappa^{2}\;\mathrm{ds}$$

and

(11) $$U_{\mathrm{shear}}=\frac{1}{2}\int_{0}^{L}\mathrm{kGA}\;\gamma_{s}^{2}\;\mathrm{ds}$$

Here *κ* is the incremental section-rotation gradient, and *γ_s_* is the local Timoshenko shear strain. The end rotations of each free web are zero, its vertical end displacement difference is zero, and its horizontal end displacement difference is Δ. Internal displacements and rotations are free to equilibrate. Flat segments at the extreme heights follow the rigid faces and store no strain energy in this loading case.

The flexibility integral is evaluated piecewise, and angular junctions enforce continuity of displacement and incremental section rotation. The initial tangent direction may jump at an ideal rigid corner without assigning an infinite deformation-induced bending strain to the joint. This explicit frame treatment is distinct from differentiating a smooth-profile formula through a corner.

A pointwise smooth-profile expression requiring *θ′* must be restricted to segments for which the tangent is regular and the relevant curvature energy is finite. For a finite-radius bend, *t_f_*/*R_min_*≪1 is a physical slenderness requirement, not an experimentally calibrated error threshold. The reference rounded profile has *t_f_*/*R_min_* = 0.75; numerical convergence for that profile therefore does not establish local curved-beam accuracy.

The equivalent homogenized response was again determined from the energy-equivalence condition

(12) $$U=\frac{1}{2}K_{\mathrm{xz}}^{*}\Gamma^{2}p$$

The energy is evaluated after equilibrium relaxation. With Δ = *Γh_c_*, the equivalent core modulus follows from the same normalization:

(13) $$G_{\mathrm{xz}}^{*}=\frac{K_{\mathrm{xz}}^{*}}{h_{c}}$$

For reproducible solution, let **q** = (*F_x_*,*F_z_*,*M*) be the tip resultants of a free web ending at (*x_B_*, *z_B_*). At a point with tangent (*t_x_*, *t_z_*), define **a***_N_* = (*t_x_*,*t_z_*,0), **a***_V_* = (−*t_z_*,*t_x_*,0), and **a***_M_* = (−(*z_B_* − *z*),*x_B_* − *x*,1). The web flexibility is $F=\int [{a_{N}}^{T}a_{N}/(\mathrm{EA})\;+{a_{V}}^{T}a_{V}/(\mathrm{kGA})+{a_{M}}^{T}a_{M}/(\mathrm{EI})]\;\mathrm{ds}$. Solving **Fq** = (Δ,0,0)^T^ gives the reaction and energy. For two symmetric webs per pitch, *G_xz_* = 2*h_c_*(**F**^−1^)_11_/*p*. The scripts implement this integral directly.

The affine and relaxed models differ in admissible internal displacements and in retained energy terms. Adding positive bending or shear energy to an unchanged displacement field cannot explain a reduction in stiffness. The reduction for a curved web is instead associated with equilibrium relaxation; for a straight inclined web the affine axial contribution is already large.

### 2.4. Integration Along the Exact Corrugated Path

The force-flexibility calculation uses the actual parametric centerline. This permits vertical segments and curved connections to be integrated without treating the entire path as a single-valued graph. It is an integration strategy for Section 2.3, not an additional independently normalized model.

For portions of the profile admitting a graph z(x), the arc length is

(14) $$L=\int_{0}^{p}\sqrt{1+{\left(z^{\prime }\left(x\right)\right)}^{2}}\;\mathrm{dx}$$

The transformation between the projected coordinate *x* and the arc coordinate *s* is therefore given by

(15) $$\mathrm{ds}=\sqrt{1+{\left(z^{\prime }\left(x\right)\right)}^{2}}\;\mathrm{dx}$$

Using this representation, the total strain energy of the flute wall may again be expressed as the sum of axial, bending, and local shear contributions:

(16) $$U=\frac{1}{2}\int_{0}^{L}\left(\mathrm{EA}\;\varepsilon_{s}^{2}+\mathrm{EI}\;\kappa^{2}+\mathrm{kGA}\;\gamma_{s}^{2}\right)\;\mathrm{ds}$$

The local fields in this expression satisfy the equilibrium and end conditions specified in Section 2.3. Initial geometric curvature is not substituted for deformation-induced curvature change.

The equilibrium energy uses the same normalization as Equation (12):

(17) $$U=\frac{1}{2}K_{\mathrm{xz}}^{\left(\mathrm{arc}\right)}\Gamma^{2}p$$

The arc notation denotes the same relaxed core modulus evaluated along the exact path:

(18) $$G_{\mathrm{xz}}^{\left(\mathrm{arc}\right)}=\frac{K_{\mathrm{xz}}^{\left(\mathrm{arc}\right)}}{h_{c}}$$

Equation (7) contains the arc-length Jacobian for its affine field. Rewriting that same integral in arc coordinates must preserve its value. Accordingly, the result tables report one affine estimate for both coordinate representations.

Each smooth segment is integrated with 96-point Gauss–Legendre quadrature in its own regular parameter. Repeating the calculation with 192 points changes the specified moduli by less than 10^−10^ in relative terms. The sine length and Equation (7) are also checked by independent adaptive integration in *x*, and the circular, triangular, and rectangular lengths are checked against closed forms.

The exact-path flexibility result is the numerical reference for FE discretization error. Agreement verifies two implementations of the same beam model; it cannot establish the accuracy of its contact or constitutive assumptions for manufactured board.

### 2.5. Finite Element Reference Model

For xz shear, the same explicitly defined core is discretized by two-node planar frame elements with axial and Timoshenko bending–shear response. Each straight element uses its exact static stiffness, with $\psi =12\mathrm{EI}/(\mathrm{kGA}l_{e}^{2})$; this avoids the shear locking associated with fully integrated equal-order displacement elements. Nodes are placed at every geometric junction and contact boundary.

The nodal equilibrium of the discretized system is expressed in the standard form

**Ku** = **f** (19)

where **K** is the global stiffness matrix, **u** is the vector of nodal degrees of freedom, and **f** is the external load vector associated with the imposed macroscopic shear distortion.

The full period starts and ends at an upper contact, with a lower contact at half pitch. At all upper/lower contact nodes, *u_x_* = ±Δ/2, *u_z_* = 0, and *ϕ* = 0, where Δ = 0.001 mm. The full horizontal plateaus are constrained when present. Opposite end nodes therefore have equal translations and rotations, satisfying periodicity for this cell origin. Free interior degrees of freedom are solved by sparse elimination. No vertical displacement jump is imposed between equal-height end nodes.

The total internal strain energy of the discretized model was computed as

(20) $$U=\frac{1}{2}u^{T}\mathrm{Ku}$$

and equated to the macroscopic shear energy of the equivalent unit cell,

(21) $$U=\frac{1}{2}K_{\mathrm{xz}}^{\left(\mathrm{FE}\right)}\Gamma^{2}p$$

which gives the effective transverse shear stiffness $K_{\mathrm{xz}}^{\left(\mathrm{FE}\right)}$. The corresponding effective modulus was defined as

(22) $$G_{\mathrm{xz}}^{\left(\mathrm{FE}\right)}=\frac{K_{\mathrm{xz}}^{\left(\mathrm{FE}\right)}}{h_{c}}$$

The frame and the separate scalar *yz* model were evaluated with 48, 96, 192, 300, 384, 768, and 1536 elements per full pitch. Relative error is measured against the exact-path flexibility or membrane solution, rather than against only the next mesh. At 300 elements, the maximum frame error is 0.11928%; at 1536, it is 0.00492%. The maximum scalar *yz* error at 1536 elements is 0.00071%. These values concern the global stiffness of the stated reduced models, not continuum stresses at corners.

It should be emphasized that the present finite element model represents a numerical realization of the same beam-type mechanical idealization used in the analytical approaches. Consequently, it serves as a consistency and comparison tool rather than as a full continuum-scale model capable of resolving local adhesive failure, thickness-wise heterogeneity, or detailed stress concentrations at sharp corners.

The MATLAB 24.1.0.3050227 (R2024a) Update 8 implementations pass 71 mechanical checks, covering rigid-body modes, analytical beam solutions, equilibrium, reaction energy, periodicity, and linear scaling. Seven closed-form benchmarks are reported in Appendix B. Table 1 distinguishes the mechanical descriptions from their numerical solution routes.

### 2.6. Board-Level Effective Transverse Shear Stiffness

The results presented in subsequent sections are core properties under prescribed rigid face motion. For a separate ideal uniform-traction series estimate involving deformable layers, the layer displacement differences, rather than the engineering strains alone, must be added.

For a simple series-compliance estimate, consider uniform shear traction through the layer stack and perfect displacement compatibility at the interfaces. The relative face displacement is the sum of the layer displacements; the engineering strains themselves must therefore be weighted by layer thickness:

*hΓ_tot_* = *h_c_Γ_f_* + *t_l_Γ_l_*_,1_ + *t_l_Γ_l_*_,2_ (23)

Using the same shear traction in each layer gives

(24) $$\frac{h}{G_{\mathrm{eq}}}=\frac{h_{c}}{G_{f}}+\frac{t_{l}}{G_{l,1}}+\frac{t_{l}}{G_{l,2}}$$

where *G_f_* is the effective core shear modulus and *G_l_*_,1_ and *G_l_*_,2_ are the liner shear moduli in the selected direction. This relation is an ideal uniform-traction estimate, not a general solution for the nonuniform shear field of a sandwich plate.

For identical liners with modulus *G_l_*, the estimate becomes

(25) $$\frac{1}{G_{\mathrm{eq}}}=\frac{1}{h}\;\left(\frac{h_{c}}{G_{f}}+\frac{2t_{l}}{G_{l}}\right)$$

The corresponding board stiffness per unit width and the effective board modulus are related by

(26) $$G_{\mathrm{eq}}=\frac{K_{\mathrm{eq}}}{h}$$

where *h* = *h_c_* + 2*t_l_* is the nominal board thickness. *G_f_* and *G_l_* must refer to compatible shear directions and constitutive definitions. The paper in-plane modulus *G*_12_ cannot automatically be assigned as a liner through-thickness shear modulus.

This relation requires independently specified liner transverse shear data. These data are not supplied here, so the relation is not used to transform the computed core values into whole-board predictions.

For plate or shell simulation, the homogenized coefficients must reproduce the appropriate board-level shear energy and account for the assumed through-thickness deformation. The present rigid face cell omits liner bending, adhesive compliance, contact offsets, and shear correction effects associated with the full sandwich; its core coefficients should not be inserted as validated whole-board properties without that additional modeling step.

### 2.7. Membrane-Path Model for Shear Along the Flute Axis

**Figure 3:**
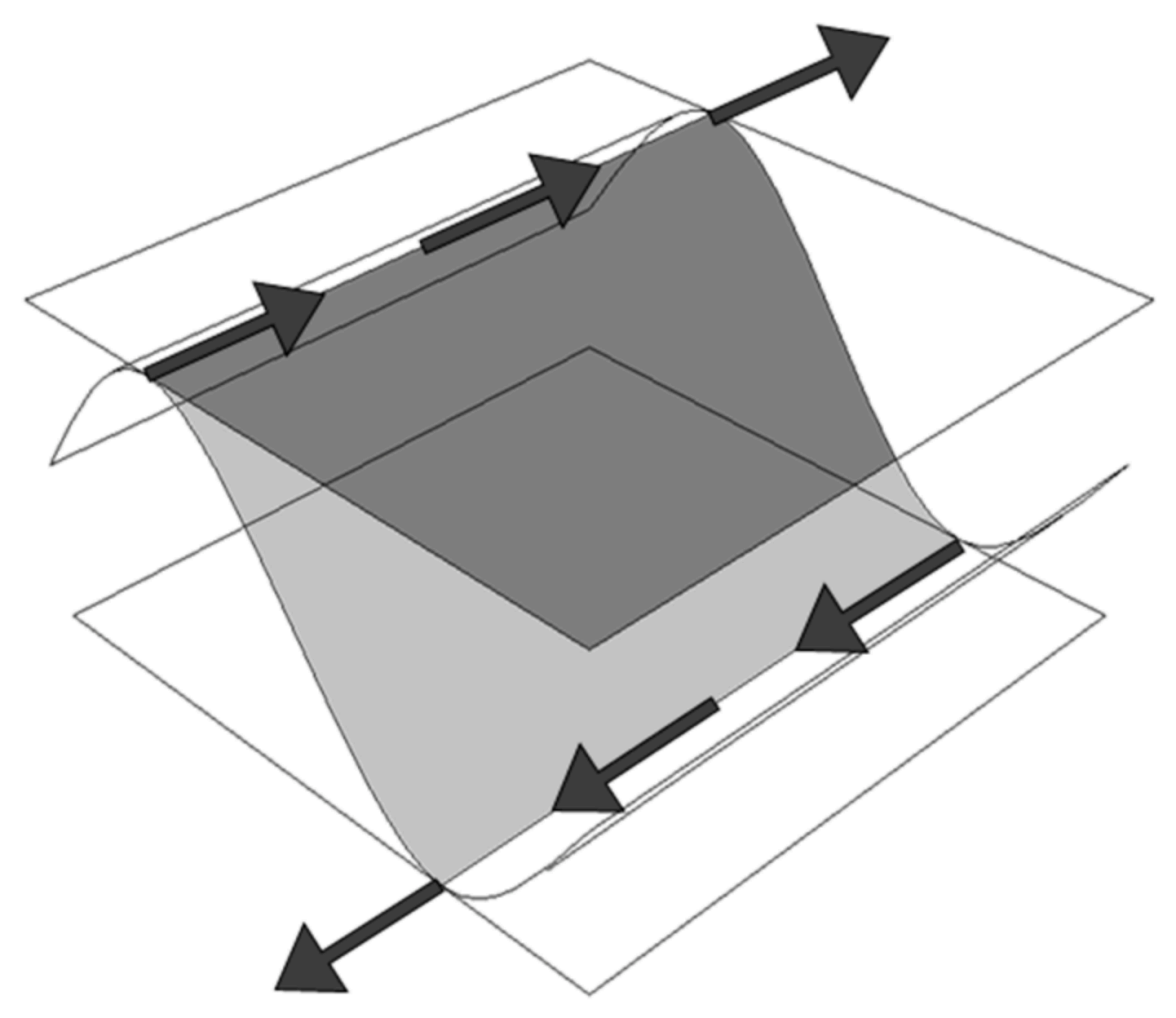
Schematic transfer of shear parallel to the flute axis. The membrane-path model uses two identical unbonded webs per period, joined to rigid translating contact levels; bonded horizontal plateaus do not contribute free-path compliance.

Shear in the *yz* plane is evaluated separately from the planar frame (see Figure 3). A scalar displacement *u_y_*(*s*) acts along the flute axis; the free web stores membrane shear energy $\left(G_{12}t_{f}/2\right)\int {\left(du_{y}/\mathrm{ds}\right)}^{2}\mathrm{ds}$. This is a reduced shear-transfer description related to the corrugated-core load-path concepts discussed in [24], with the present branch counting and boundary conditions stated explicitly.

For a homogeneous free web of length *ℓ*, equilibrium gives a constant derivative d*u_y_*/*ds* = Δ/𝓁 and branch stiffness *G*_12_*t_f_*/𝓁. Two such branches occur per full pitch, so the core modulus is

(27) $$G_{\mathrm{yz}}=\frac{2h_{c}t_{f}}{pl}G_{12}$$

where 𝓁 is the unbonded length from upper to lower contact and *h_c_* is the separation of the contact centerline levels. For profiles without bonded horizontal plateaus, 𝓁 = *L*/2. For flat-topped profiles, the bonded plateau lengths are excluded. The two-branch factor and core-height normalization differ from an expression written using total board thickness and the complete half-wave length; these are different definitions and must not be interchanged.

The dimensionless geometric coefficient for this particular membrane-path model is

(28) $$\eta =\frac{2h_{c}t_{f}}{pl}$$

It measures the shear transfer of two unbonded branches under the imposed relative displacement; it is not a general material-efficiency index.

A separate one-degree-of-freedom-per-node FE model uses element stiffness (*G*_12_*t_f_*/𝓁*_e_*) [1,−1;−1,1] and prescribed *u_y_* = ±Δ/2 at the contact nodes. Its energy is normalized by the same core volume p *h_c_* per unit width. This scalar membrane model is not a yz solution extracted from the *xz* planar frame.

The formulation excludes out-of-plane bending of the paper strip, finite-width effects, liner deformation, and adhesive compliance. Its agreement with the scalar FE solution is a numerical verification of the path model, not an experimental validation of corrugated-board shear.

### 2.8. Flute Profiles and Parametric Study Design

**Figure 4:**
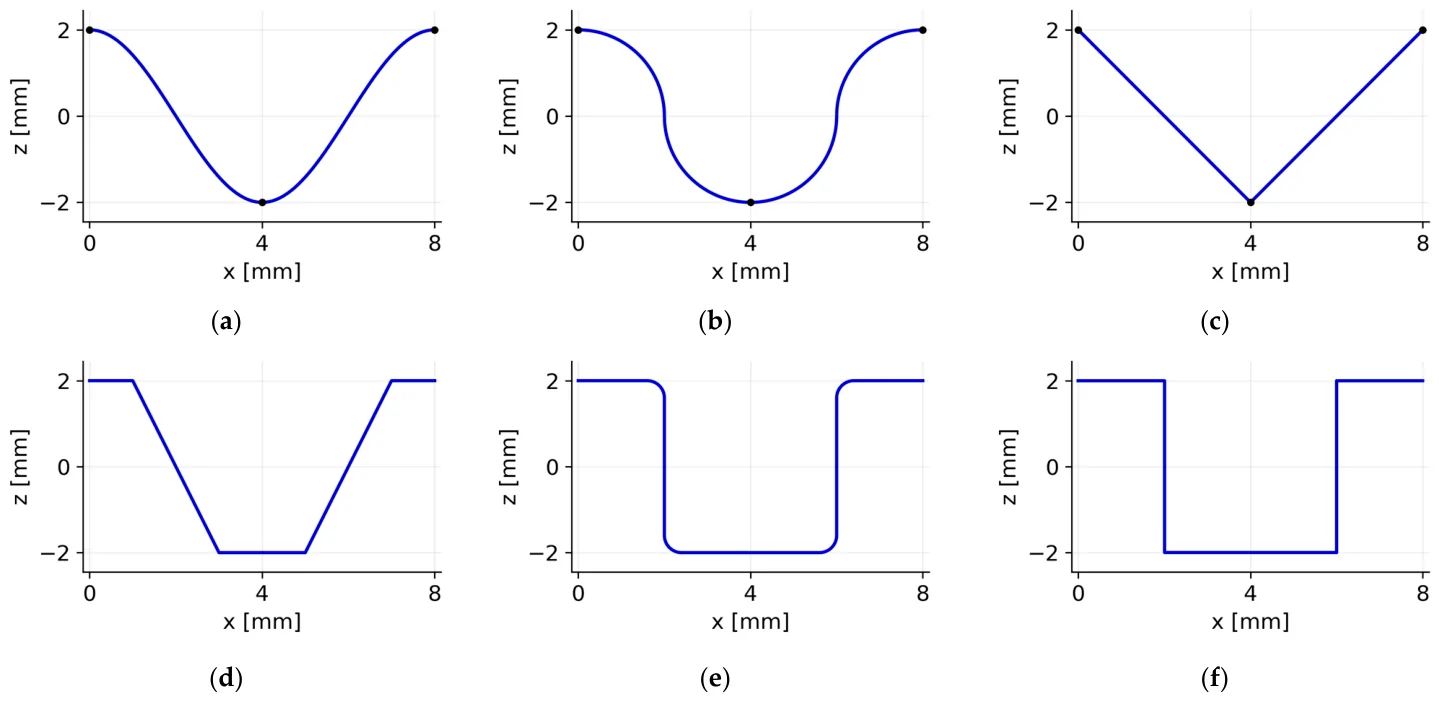
Flute centerlines analyzed in this study, all with p = 8 mm and *h_c_* = 4 mm: (**a**) sinusoidal; (**b**) circular-arc; (**c**) triangular; (**d**) trapezoidal with total plateau fraction α = 0.50; (**e**) rounded-rectangular with r = 0.40 mm; (**f**) rectangular. Black points mark isolated constrained contacts; horizontal plateaus at the extreme heights are fully constrained. The geometric definitions and contact convention are explicit assumptions of the model.

Six explicitly defined centerlines are used: sinusoidal, circular-arc, triangular, trapezoidal, rounded-rectangular, and rectangular. Figure 4 shows their common pitch and core height. The complete parametric functions are supplied in the scripts and summarized in Appendix C.

The sinusoid is the reference. The circular-arc profile is made from tangent-connected quarter circles at *h_c_*/*p* = 0.5; independent changes in height or pitch produce an arc-derived elliptical family. The trapezoid has total horizontal-plateau fraction *α* = 0.50, and the rounded rectangle uses *r*/*h_c_* = 0.10. These shape parameters define the comparisons in this study and are not fitted to a target stiffness. Table 2 gives the input values and the integrated sine length.

The computations use *E_MD_*, *G*, *G*_12_, and k. *E_CD_* and ν are retained as contextual source inputs but are not used by these directional reduced models. The value *h* = 4.60 mm is nominal; contact offsets associated with the finite flute thickness are not modeled.

The take-up factor introduced in Section 2.1,

(29) $$T=\frac{L}{p}$$

was used as an auxiliary geometric indicator linking flute shape with both material consumption and structural efficiency.

The local study varies *h_c_*, p, or *t_f_* by −10%, −5%, 0%, +5%, and +10%, with all other independent parameters fixed. The plateau fraction and *r*/*h_c_* are held constant. Each waveform is evaluated separately; no average over physically different models is formed. Both core modulus G and rigidity *K* = *Gh_c_* are recorded.

To facilitate comparison between different variables, the normalized sensitivity measure

(30) $$S=\frac{\Delta K/K_{0}}{\Delta q/q_{0}}$$

was adopted, where *K*_0_ and *q*_0_ denote the reference stiffness and reference geometric parameter, respectively, while Δ*K* and Δ*q* are their corresponding variations.

Sensitivity is local to the specified geometry and contact convention. A change in height affects modulus normalization as well as the mechanical response, so *K*/*K*_0_ and *G*/*G*_0_ must be distinguished. The purpose is to examine the computed trends, including cases that contradict a simple monotonic design rule.

Complete Python 3.12.14 with NumPy 2.3.5, SciPy 1.17.0, pandas 2.2.3 i Matplotlib 3.10.8 and MATLAB implementations include geometry functions, direct formula evaluation, complementary-energy integration, sparse FE models, verification checks, analytical benchmarks, the full mesh sequence, local sensitivity data, and plotting code. Dimensional and normalized results and the numerical values underlying every result figure are supplied as CSV files. The execution environments and comparison of the independently generated datasets are documented in the computational supplement.

## 3. Results and Discussion

### 3.1. Comparison of Modeling Approaches

The comparison first evaluates the fully specified affine equations and then the equilibrium frame and membrane-path models. The explicit profile parameters and face constraints are essential parts of the definition of each calculation.

For the sine reference, direct integration gives *L* = 11.71 mm and *T* = 1.46. Equation (7) gives *G_xz_* = 88.01 MPa, whereas the relaxed frame gives 29.93 MPa. The triangular affine-to-sine ratio is 1.205, and the exact rectangular affine contribution is zero. Table 3 gives the dimensional values and the separate normalized frame response.

The response depends strongly on the allowed deformation. A straight triangular web carries much of the imposed shear through axial extension, whereas curved or near-vertical paths permit a larger bending contribution. The affine axial estimate therefore cannot be treated as a uniformly accurate approximation to the relaxed frame across all families.

The yz results in Table 4 follow from the separately defined membrane-path model. Their ranking differs from the xz frame results in Table 3. The sinusoidal dimensional reference is *G_yz_* = 40.99 MPa.

The maximum frame FE discrepancy at the final mesh is 0.00492%, occurring for the rounded-rectangular profile. Straight polygonal free webs agree to numerical roundoff when all junctions are represented. This verifies the stiffness solution of the reduced models; it does not imply comparable accuracy for local stresses or real board behavior.

**Figure 5:**
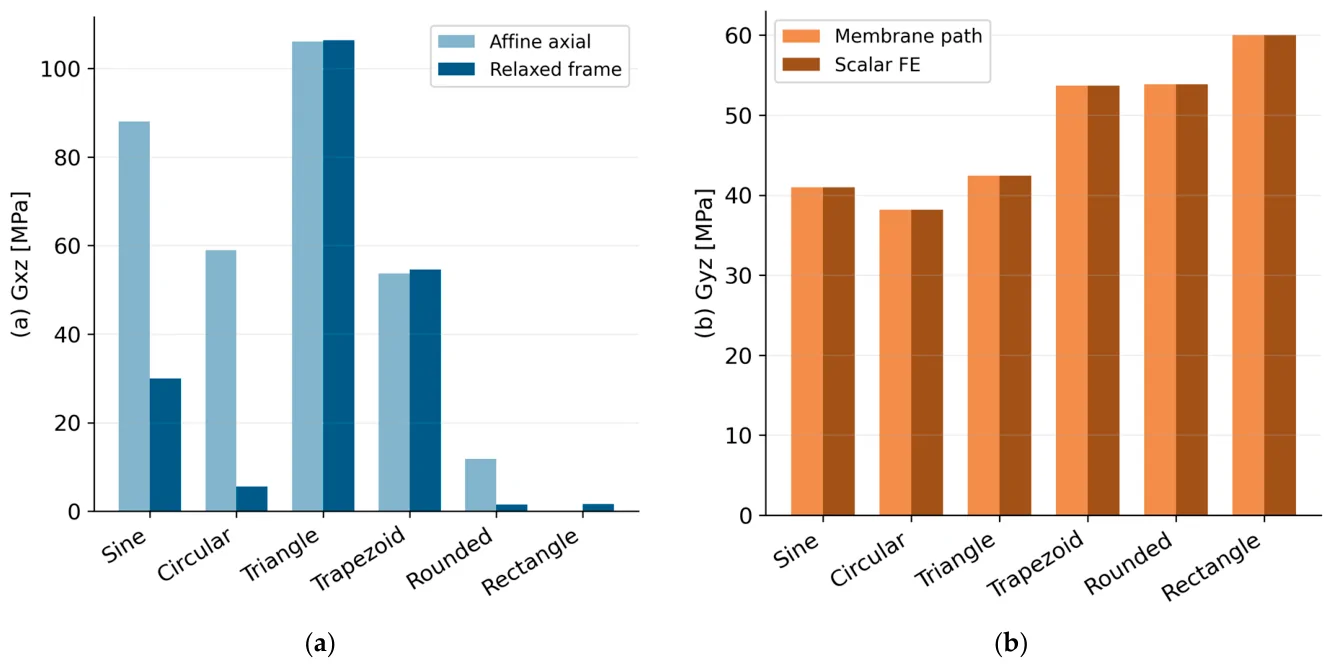
Computed directional core moduli. (**a**) Affine axial Equation (7) and relaxed frame *G_xz_*; (**b**) exact membrane-path *G_yz_* and scalar FE solution. Frame values span 1.524–106.360 MPa; membrane values span 38.197–60.000 MPa. Each model has the contact and normalization assumptions stated in Section 2; no cross-model average is used.

Figure 5 reports dimensional results rather than only ratios. The broad xz spread shows that a change in waveform can produce substantial differences even at common nominal pitch and height under the specified contact convention.

The comparison establishes directional and model-dependent behavior. Numerical agreement between the force method and FE checks implementation, while the substantial affine–frame differences quantify the effect of changing the kinematic model within the stated idealization.

### 3.2. Influence of Flute-Profile Family

Waveform family has a substantial influence on the xz frame results. Relative to the sine, the normalized values range from approximately 0.051 for the rounded rectangle to 3.55 for the triangle. This comparison also includes changes in bonded plateau length, which are inherent in the chosen contact convention and must not be interpreted as curvature effects alone.

The triangular frame response is largely axial: approximately 0.27% of its total strain energy is bending energy under the imposed loading. The corresponding bending fractions are about 50.58% for the sine, 83.18% for the circular-arc profile, 86.20% for the rounded rectangle, and 96.74% for the rectangle. These values explain why relaxation affects the families differently.

In the yz path model, the relevant compliance is the unbonded path length. The circular-arc family has the lowest computed modulus, 38.19719 MPa. The rectangular family reaches 60 MPa because each vertical free web has length *h_c_*, while its horizontal plateaus follow the rigid contact surfaces. This ranking is conditional on full plateau bonding.

A rectangular path illustrates the need to identify the mechanism: its affine axial energy is zero, its xz frame stiffness is positive through bending and local shear, and its yz membrane shear stiffness is positive. None of these observations is inconsistent once the different strain fields are stated.

The calculated ranges do not support general dominance of geometric proportions over waveform family. The shape comparison and the ±10% parameter study cover different design changes, and their numerical hierarchy depends on direction, mechanism, and contact conditions.

Writing *z* = *h_c_f*(*x*/*p*) shows how *h_c_*/*p* scales slope, but it does not establish dominance over a change in f itself. In the affine estimate *ε_s_* = *Γ* cos *θ* sin *θ*, both horizontal and vertical segments have zero axial contribution. In the relaxed model, the full compliance and contact constraints control load transfer. The energy partitions reported above provide a mechanism-based interpretation of the differences between flute profiles.

**Figure 6:**
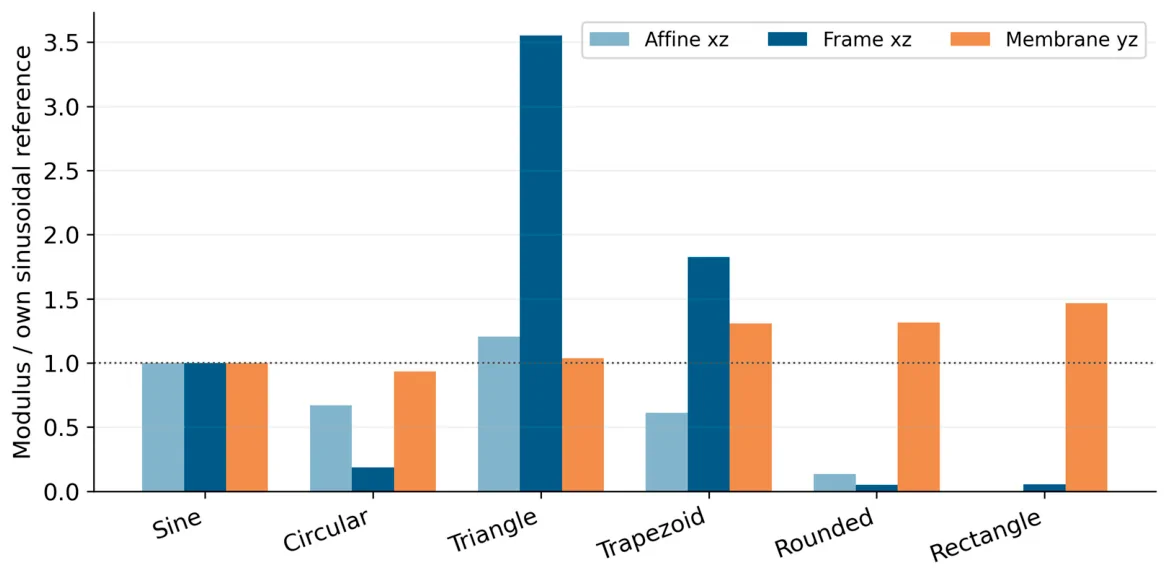
Normalized computed response by waveform. The xz frame range is 0.051–3.553, and the yz membrane range is 0.932–1.464, each relative to its own sinusoidal value. The affine xz estimate is shown separately. These are calculated extrema of the reported datasets.

Figure 6 therefore shows separate normalized results for each mechanical description. It does not average them into a single stiffness score. A material-efficient or manufacturable optimum would require additional constraints and performance measures that are not established here.

### 3.3. Parametric Sensitivity to Flute Height, Pitch, and Wall Thickness

**Figure 7:**
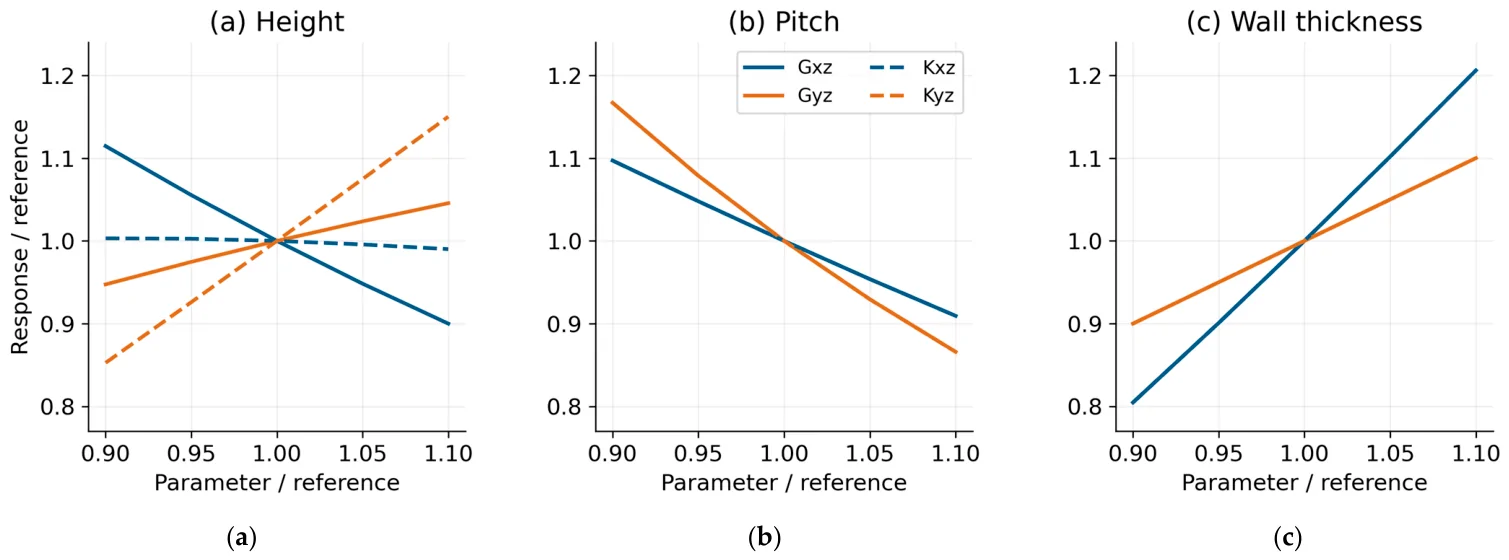
Sinusoidal local sensitivity for (**a**) *h_c_*, (**b**) *p*, and (**c**) *t_f_* over 0.9–1.1 times the reference parameter. Curves give *G_xz_*/*G_xz_*_,0_ and *G_yz_*/*G_yz_*_,0_ (solid) and *K_xz_*/*K_xz_*_,0_ and *K_yz_*/*K_yz_*_,0_ (dashed). At *h_c_*/*h_c_*_,0_ = 1.1, the frame modulus ratio is 0.9001, while its rigidity ratio is 0.9901. These response ratios are distinct from the coefficient in Equation (30).

The local parameter effects also depend on the response definition. Figure 7 shows the sinusoidal reference; complete data for all six families are supplied with the scripts. Solid curves represent modulus ratios and dashed curves rigidity ratios, preventing a height-dependent change in normalization from being mistaken for a change in load-transfer stiffness.

Height, pitch, and wall thickness were varied independently over ±10%. All reported curves use the same explicit geometry construction and contact convention as the baseline models.

Increasing sinusoidal height by 10% lowers the frame *G_xz_* by 9.994%, while *K_xz_* decreases by only 0.994%. Over the same change, the membrane *G_yz_* rises by 4.556% and *K_yz_* by 15.012%. The height dependence is therefore directional and differs between modulus and rigidity.

Increasing pitch by 10% reduces the sinusoidal frame *G_xz_* by 9.058% and the membrane *G_yz_* by 13.407%. These are local trends for the specified families and constraints, not universal relations for any change in flute geometry.

Increasing wall thickness by 10% raises the sinusoidal frame *G_xz_* by 20.604%, whereas the membrane *G_yz_* increases by 10%. The stronger frame dependence reflects the mixture of EA and EI contributions; the affine axial and membrane formulas remain linear in *t_f_*.

The computed thickness sensitivity can be interpreted through the energy partition, but comparing a ±10% dimensional perturbation with a full change in waveform does not establish a universal ranking of design variables.

The results do not support a general recommendation that increasing flute height is the most effective means of raising both transverse shear moduli. An appropriate design choice must identify the required shear direction, whether G or K is relevant, and the accompanying material, bonding, and stability constraints.

### 3.4. Structural Efficiency and Take-Up Factor

The normalized stiffness comparison can also be considered alongside the take-up factor T = L/p, which measures the amount of medium required per projected pitch.

**Figure 8:**
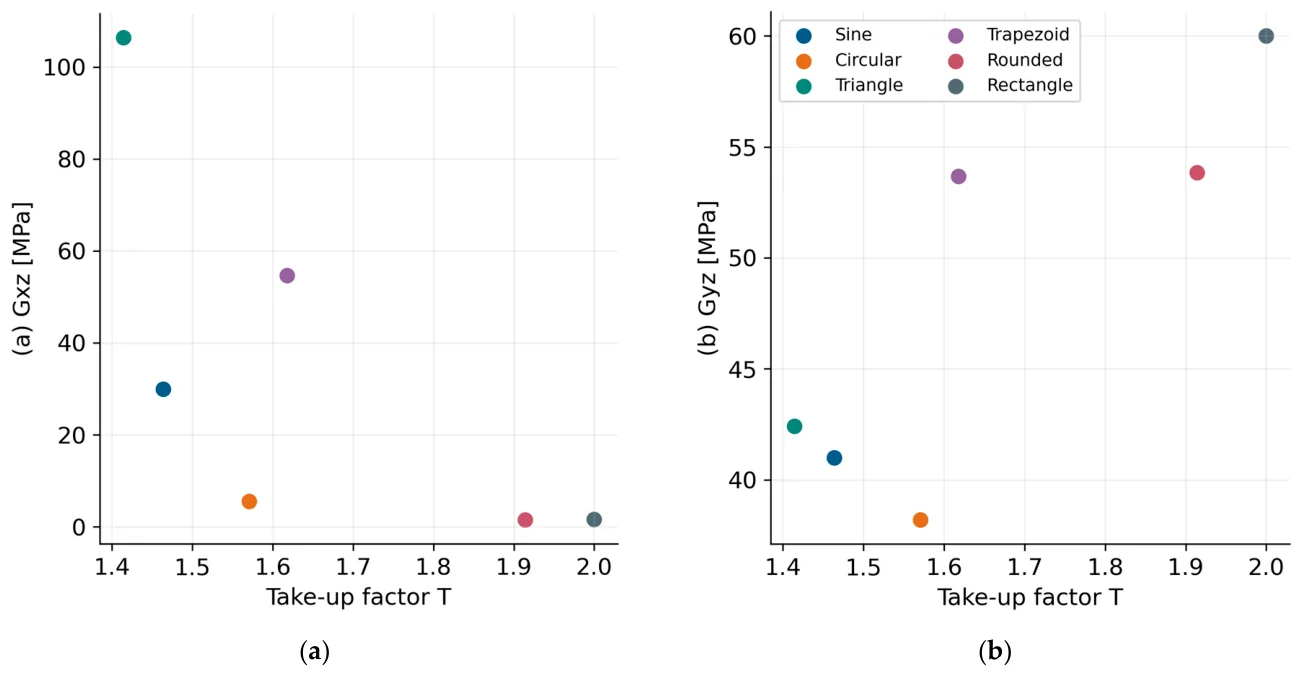
Core modulus versus take-up factor for the explicit geometries: (**a**) relaxed frame *G_xz_*; (**b**) membrane-path *G_yz_*. T ranges from 1.414 to 2.000. The two directions are shown separately; no unvalidated composite efficiency score or favorable-region boundary is assigned.

Figure 8 compares directional specified moduli with the computed take-up factor. At common p and *h_c_*, the sine has *T* = 1.464, the triangle 1.414, and the rectangle 2.000. Material usage is therefore different even though nominal cell dimensions are equal.

The triangle has the smallest take-up factor among these definitions and the largest xz frame modulus. The rectangle uses more medium and has a much smaller xz frame modulus, while its yz membrane modulus is largest under full plateau bonding. A single stiffness-versus-material ranking would hide this directional distinction.

The take-up factor records the unfolded centerline length and is proportional to medium mass at fixed density and thickness. It contains no direct information about bond quality, nonlinear strength, local buckling, or manufacturing tolerances.

For profiles with plateaus, increasing total material path need not increase the free shear-transfer length by the same amount because bonded segments follow the rigid faces. This explains why T alone does not determine the membrane response.

A defensible efficiency assessment must specify the loading direction and include the additional constraints relevant to production and use. The present results provide mechanical comparisons within the stated idealization only.

### 3.5. Design Implications and Practical Interpretation

Model choice, contact convention, waveform, and geometric proportions can all influence the response. Their relative importance depends on the mechanism and comparison range, so no universal hierarchy of these factors follows from the present results.

The primary numerical reference uses 1536 elements per period. Including 300 elements in the convergence sequence demonstrates the size of the stiffness error at a commonly plausible intermediate discretization, rather than treating element count alone as evidence of convergence.

**Figure 9:**
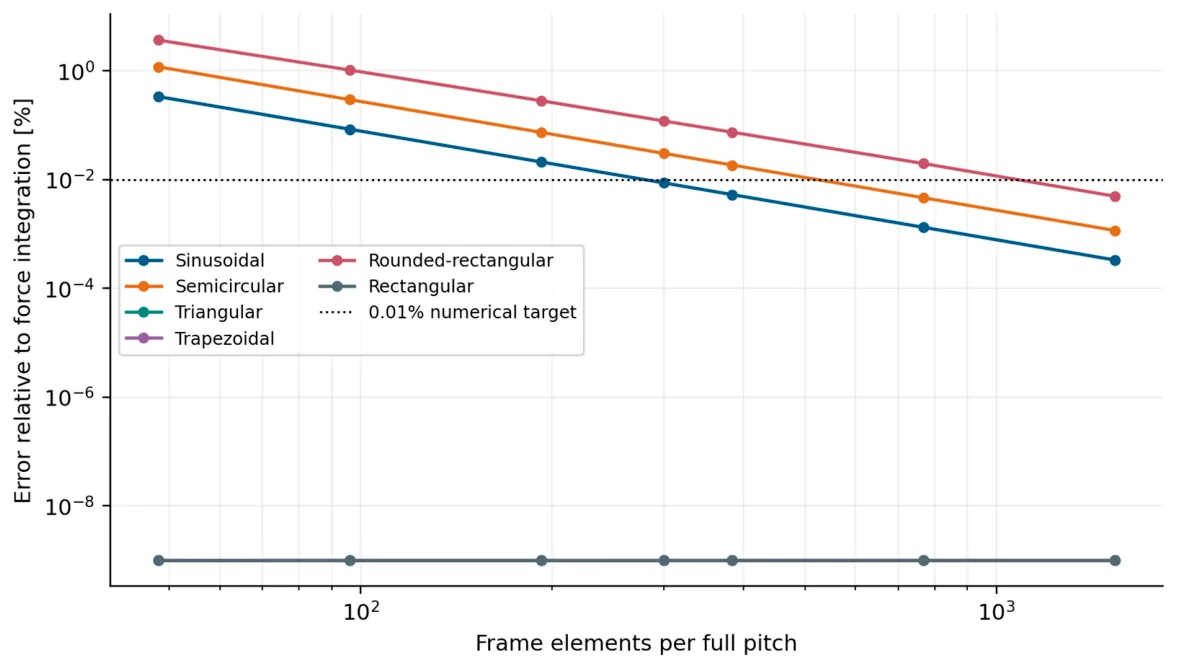
Relative xz frame stiffness error against exact-path force-flexibility integration for 48, 96, 192, 300, 384, 768, and 1536 elements per pitch. The largest error falls from 0.1193% at 300 elements to 0.0049% at 1536. The dotted line marks a 0.01% numerical target. Errors below 10^−9^% are plotted at that floor for logarithmic display. The plot does not represent continuum corner-stress convergence or experimental accuracy.

Figure 9 gives measured numerical convergence errors. It addresses discretization of the reduced models; manufacturability and robustness are not quantified by this calculation.

Numerical convergence of a sharp-junction frame does not remove uncertainty in the physical joint model. All corners are ideal rigid joints, and all face contacts are ideal. For the rounded reference, *t_f_*/r = 0.75, which is not a small curved-beam slenderness ratio. Its value is therefore a numerical model result with a particularly limited local physical interpretation.

The code also provides a corner-radius diagnostic over *r* = 0.05 ÷ 1.20 mm. It measures response changes inside the same beam idealization; it does not establish a model-error threshold against a shell, solid, or experimental reference.

The computational framework is useful for transparent comparative calculations and for identifying assumptions that need further evidence. It does not establish an industrial optimum or a validated package-level constitutive model.

### 3.6. Experimental Context and Limitations

The experimental comparison uses published data. Popil et al. [6], Table 1, provide measurements for the conventional C-flute specimen IEC4226C: transverse shear rigidities *R*_55_ = 9760 N/m and *R*_44_ = 54,300 N/m before nip crushing, falling to 3410 N/m and 23,300 N/m afterwards. These are board-level directional rigidities, not moduli in MPa.

For dimensional context, the present idealized sinusoidal core gives *K_xz_* = 119.73 kN/m and *K_yz_* = 163.97 kN/m. This juxtaposition is not a matched prediction of specimen IEC4226C: constituent properties, geometry, thickness convention, conditioning, face compliance, and test-based shear definition have not been matched. No percentage prediction error or calibrated accuracy range is inferred. The appreciable scale difference reinforces the need to distinguish rigid face core calculations from experimentally identified whole-board stiffness.

For conventional corrugation, x corresponds to MD and y to CD; the sinusoidal-core ordering *K*_xz_ < *K*_yz_ is consistent with the measured ordering *R*_55_ < *R*_44_ [6], although the absolute values differ substantially. Published shell simulations [26] also show that transverse shear stiffness depends on flute discretization and element formulation, supporting the need for the present mesh-refinement checks. Those simulations allow liner deformation, whereas the present core model imposes rigid face contacts; their stiffness values therefore represent different mechanical constraints. Imperfection-aware studies [27,28] additionally account for mechanisms excluded here. This comparison provides qualitative support for the mechanistic interpretation; quantitative predictive accuracy for manufactured board remains unverified.

The model assumes small strains, linear elasticity, explicit ideal centerlines, and perfect contact. It excludes adhesive thickness and compliance, bond defects, flute asymmetry, contact offsets, forming damage, local buckling, and moisture-dependent material changes. These effects can alter absolute stiffness and profile rankings. The assigned constants are not a measured environmental characterization.

The results apply to an undeformed, undamaged reference state. They are not tangent moduli for pre-compressed or crushed board and do not cover large rotations. Those applications require the actual deformed geometry, prestress, and incremental constitutive response. At package scale, a homogenized description also requires a response length scale large relative to pitch; creases, edges, concentrated loads, and local joints can require a resolved model.

## 4. Conclusions

Separately defined and reproducible *xz* and *yz* calculations give distinct directional results. A sinusoid with *p* = 8 mm and *h_c_* = 4 mm has *L* = 11.71 mm and *T* = 1.46; its affine axial, relaxed frame, and membrane-path responses are mechanically different.

Independent force-flexibility integration and frame FE assembly agree within 0.0049% at 1536 elements per pitch. The analogous membrane discrepancy is at most 0.0007%. These checks establish numerical consistency inside the reduced models, not independent physical or experimental validation.

Waveform changes produce substantial differences in the xz response, and the yz ranking is different. A general assertion that waveform family is secondary to geometric proportions is not supported by this comparison. The contact-length convention is part of the stated result.

For the sinusoidal reference, increasing height by 10% decreases frame *G_xz_* by approximately 9.99% while changing *K_xz_* by only −0.99%; it increases membrane *G_yz_* by about 4.56%. Modulus and rigidity, and the two shear directions, must therefore be distinguished when interpreting geometric sensitivity.

The take-up factor describes medium consumption and complements the stiffness comparison. It is not itself a mechanical efficiency measure, and equal nominal dimensions do not ensure equal material mass across waveform families.

Piecewise integration and explicit rigid-junction conditions permit sharp profiles to be solved within a frame idealization. Smooth pointwise curvature formulas cannot simply be applied across a tangent discontinuity. Neither mesh convergence nor a finite corner radius supplies a universal physical prediction-error bound.

The reported properties apply to the idealized core under rigid translating contact constraints. They are not whole-board properties for deformable liners and should be used in packaging simulations only with a compatible energy definition and an assessment of the omitted face and bonding mechanisms.

The study is analytical and numerical. Manufactured imperfections, adhesive compliance, moisture, local buckling, large rotations, pre-compression, and material nonlinearity remain outside the model. A matched experimental validation and a calibrated accuracy estimate remain unavailable.

## Figures and Tables

**Table 1:** Summary of modeling approaches used in the study and their respective roles within the comparative framework.

Description/Route	Mechanisms	Geometry	Relative Effort	Role
Affine axial estimate	Prescribed axial strain	Exact centerline	Low	Kinematic comparison
Relaxed frame	Axial, bending, local shear	Piecewise centerline	Moderate	xz equilibrium model
Force integration	Same frame mechanics	Exact parametric path	Low	Numerical reference
Frame FE	Same frame mechanics	Straight segment mesh	Higher	xz discretization check
Membrane path	In-plane paper shear	Free web length	Low	Separate yz model

**Table 2:** Reference geometric and material parameters adopted in the comparative analyses.

Symbol	Description	Reference Value	Unit
*p*	Flute pitch	8.00	mm
*h_c_*	Core height (flute height)	4.00	mm
*t_f_*	Flute-wall thickness	0.30	mm
*t_l_*	Thickness of one liner	0.30	mm
*h*	Total board thickness	4.60	mm
*L*	Unfolded flute length per pitch (sinusoidal reference)	11.71	mm
*T* = *L*/*p*	Take-up factor	1.46	–
*E_MD_*	Young’s modulus in machine direction	4000	MPa
*E_CD_*	Young’s modulus in cross direction	2000	MPa
*G*	Shear modulus (generic analytical models)	800	MPa
*G*_12_	In-plane shear modulus of paper	800	MPa
*ν*	Poisson’s ratio	0.30	–
*k*	Shear correction factor	5/6	–
*N_e_*	Number of finite elements per pitch	1536	–
Δ*q*/*q*_0_	Parameter perturbation used in sensitivity study	±10%	–
*α*	Total plateau fraction for trapezoid	0.50	–
*r*/*h_c_*	Rounded-rectangle radius ratio	0.10	–
Δ	Imposed relative contact displacement	0.001	mm

**Table 3:** Computed xz core moduli in MPa. The affine column is Equation (7); the force solution uses Section 2.3, and FE uses 1536 elements per pitch. The last column is the force-solution modulus divided by its sinusoidal value of 29.93 MPa.

Profile	Affine *G_xz_* [MPa]	Force *G_xz_* [MPa]	FE *G_xz_* [MPa]	Force/Sine
Sinusoidal	88.012	29.933	29.933	1.000
Semicircular	58.905	5.584	5.584	0.187
Triangular	106.066	106.359	106.359	3.553
Trapezoidal	53.666	54.606	54.606	1.824
Rounded-rectangular	11.781	1.524	1.524	0.051
Rectangular	0.000	1.632	1.632	0.054

**Table 4:** Computed yz core moduli from the membrane-path model and its scalar FE discretization with 1536 elements per pitch. Normalization uses the exact-path sinusoidal value, 40.99213 MPa. Error is the absolute FE–analytical difference divided by the analytical value, expressed as a percentage.

Profile	Path *G_yz_* [MPa]	FE *G_yz_* [MPa]	Path/Sine	FE Error [%]
Sinusoidal	40.992	40.992	1.000	0.00004
Semicircular	38.197	38.197	0.932	0.00007
Triangular	42.426	42.42641	1.035	0.00000
Trapezoidal	53.666	53.66563	1.309	0.00000
Rounded-rectangular	53.852	53.85265	1.314	0.00071
Rectangular	60.000	60.000	1.464	0.00000

## Data Availability

The original contributions presented in this study are included in the article. Further inquiries can be directed to the corresponding authors.

## Appendix A

### Derivation of the Strain-Energy Analytical Formulation

This appendix summarizes the derivation of the simplified analytical expression used to estimate the effective transverse shear stiffness of one periodic corrugated-board segment. The derivation is based on strain-energy equivalence between the actual corrugated medium and an equivalent homogenized unit cell.

A single flute period of projected length *p* is considered. The centerline of the corrugated medium is described by the continuous function

*z* = *z*(*x*), *x* ∈ [0, *p*] (A1)

where *x* denotes the machine-direction coordinate and *z* is measured from the reference mid-plane of the board.

The infinitesimal arc-length element of the flute wall is

(A2) $$\mathrm{ds}=\sqrt{1+{\left(z^{\prime }\left(x\right)\right)}^{2}}\;\mathrm{dx}$$

which accounts for the true geometry of the corrugated path.

A macroscopic engineering shear strain Γ imposed at the homogenized scale is represented by the displacement field

*u_x_*(*x*) = *Γz*(*x*) (A3)

Here *u_x_* is the horizontal displacement and *u_z_* = 0, so *γ_xz_* = *Γ*. The local strain follows by projecting the symmetric small-strain tensor onto the centerline tangent; it is not obtained from the vertical component of the tangent alone.

The tangent vector to the flute centerline forms the local angle *θ* such that

tan *θ* = *z*′(*x*) (A4)

and therefore

(A5) $$\sin\theta =\frac{z^{\prime }(x)}{\sqrt{1+{\left(z^{\prime }(x)\right)}^{2}}}$$

With *ε_xz_* = *ε_zx_* = *Γ*/2 and the other strain components zero, the local axial strain is *ε_s_* = *Γ* cos *θ* sin *θ*, or

(A6) $$\varepsilon_{s}=\Gamma \frac{z^{\prime }(x)}{1+{\left(z^{\prime }(x)\right)}^{2}}.$$

The corresponding strain energy stored in one flute period is

(A7) $$U=\frac{1}{2}\int_{0}^{L}\mathrm{EA}\;\varepsilon_{s}^{2}\;\mathrm{ds}$$

where E is the modulus along the paper path and *A* = *t_f_* per unit width. The denominator in Equation (A6) is $1+{\left(z^{\prime }\right)}^{2}$; using its square root would not yield the energy expression below.

After substitution of Equations (A2) and (A6), the energy becomes

(A8) $$U=\frac{1}{2}\Gamma^{2}Et_{f}\int_{0}^{p}\frac{{\left(z^{\prime }(x)\right)}^{2}}{{\left(1+{\left(z^{\prime }(x)\right)}^{2}\right)}^{3/2}}\mathrm{dx}$$

The equivalent homogenized unit cell is defined through the macroscopic shear energy

(A9) $$U=\frac{1}{2}K_{\mathrm{xz}}\Gamma^{2}p$$

which yields

(A10) $$K_{\mathrm{xz}}=\frac{Et_{f}}{p}\int_{0}^{p}\frac{{\left(z^{\prime }(x)\right)}^{2}}{{\left(1+{\left(z^{\prime }(x)\right)}^{2}\right)}^{3/2}}\mathrm{dx}$$

Finally, the effective shear modulus is obtained as

(A11) $$G_{\mathrm{xz}}=\frac{K_{\mathrm{xz}}}{h_{c}}$$

The derivation applies to the axial energy of the prescribed affine shear field. It retains the core height *h_c_* in the modulus normalization and does not include microscopic relaxation, bending, local shear, adhesive compliance, or damage. For a piecewise-smooth path the axial contribution can be evaluated segmentwise; a vertical segment requires a parametric representation.

## Appendix B

### Finite Element Reference Solution and Numerical Procedure

The finite element model provides a numerical comparison within the same beam-type idealization. It is not an independent validation of the constitutive assumptions or a three-dimensional representation of the paper and adhesive.

The xz model uses two-node extensible Timoshenko frame elements with degrees of freedom (*u_x_*,*u_z_*,*ϕ*). The static stiffness of each straight element is evaluated with $\psi =12\mathrm{EI}/(\mathrm{kGA}l_{e}^{2})$, using the standard axial block and the exact bending–shear block. All corners and contact boundaries are included as mesh nodes. The *yz* calculation is a separate scalar membrane assembly, as defined in Section 2.7.

The element stiffness matrix consisted of axial, bending, and shear contributions:

(A12) $$k^{\left(e\right)}=k_{\mathrm{axial}}^{\left(e\right)}+k_{\mathrm{bend}}^{\left(e\right)}+k_{\mathrm{shear}}^{\left(e\right)}$$

All element matrices were transformed into the global coordinate system and assembled into the global system

**Ku** = **f** (A13)

For affine-periodic kinematics consistent with *u_x_* = *Γz* and *u_z_* = 0, the displacement fluctuations are periodic. If *L* and *R* are corresponding boundaries of a full pitch, the required relations are

*u_x_*_,*R*_ − *u_x_*_,*L*_ = *Γ*(*z_R_* − *z_L_*) (A14)

*u_z_*_,*R*_ − *u_z_*_,*L*_ = 0 (A15)

*ϕ_R_* − *ϕ_L_* = 0 (A16)

At all upper/lower contact nodes, the xz constraints are *u_x_* = ±Δ/2, *u_z_* = 0, and *ϕ* = 0, with Δ = 0.001 mm. Entire extreme-height plateaus are constrained. The end nodes of the full period are both upper contacts, so their translational and rotational jumps vanish. These prescribed contact motions impose *Γ* = Δ/*h_c_*; periodicity alone does not impose shear. The internal energy is

(A17) $$U=\frac{1}{2}u^{T}\mathrm{Ku}$$

and converted into the equivalent shear stiffness:

(A18) $$K_{\mathrm{xz}}^{\mathrm{FE}}=\frac{2U}{\Gamma^{2}p}$$

with

(A19) $$G_{\mathrm{xz}}^{\mathrm{FE}}=\frac{K_{\mathrm{xz}}^{\mathrm{FE}}}{h_{c}}$$

The refinement sequence is 48, 96, 192, 300, 384, 768, and 1536 elements per pitch. Each half-period is allocated segments in proportion to arc length, with at least one element in every line or arc; the second half is mirrored. Exact junctions are retained on every mesh. The largest final frame and scalar membrane errors are 0.0049% and 0.0007%, respectively, relative to their exact-path solutions. Numerical values at every mesh are supplied as CSV data.

Each implementation passes 71 checks of rigid-body modes, analytical solutions, energy–reaction consistency, equilibrium, periodicity, and amplitude scaling. Seven explicit closed-form benchmarks are summarized in Table A1. A further 1249 numerical comparisons between independently generated MATLAB-language CSV outputs pass the documented tolerances; the largest relative difference among compared physical quantities is 4.95 × 10^−12^. These are implementation and discretization checks of idealized mechanics, not evidence of experimental accuracy.

Benchmarks B01–B03 use one straight cantilever element of length 5.7 mm, width 1 mm, and tip force 0.001 N; B01 is axial, while B02–B03 use a transverse force and free tip rotation. B04–B07 use the baseline dimensions in Table 2, 48 elements per pitch, and Δ = 0.001 mm. The frame contacts fix vertical motion and rotation; the membrane contacts prescribe transverse translation. Material constants are those in Table 2. Full definitions, closed-form equations, and full-precision comparisons are provided in the computational supplement.

**Table A1:** Closed-form verification cases. Both numerical implementations reproduce every analytical response with relative error below 10^−10^. Units belong to the stated response; these are numerical benchmarks of the reduced models.

Case	Response (Unit)	Analytical Value	Relative Error
B01	Axial cantilever u (mm)	4.75 × 10^−6^	<10^−10^
B02	Bending–shear v (mm)	0.0069	<10^−10^
B03	Tip rotation ϕ (rad)	0.0018	<10^−10^
B04	Rectangular frame *G_xz_* (MPa)	1.632	<10^−10^
B05	Triangular frame *G_xz_* (MPa)	106.36	<10^−10^
B06	Rectangular membrane *G_yz_* (MPa)	60.00	<10^−10^
B07	Triangular membrane *G_yz_* (MPa)	42.43	<10^−10^

## Appendix C

### Appendix C.1. Analytical Representation of the Compared Flute Families

All profiles have the same p and *h_c_*. The numerical full period begins at an upper contact, with a lower contact at *p*/2; the second half is the reflection of the first. Phase shifts in the sinusoid and triangular formula below do not change their geometry or results under the corresponding periodic contact sets. The trapezoidal flat fraction and rounded-corner radius are explicit inputs.

#### Appendix C.1.1. Sinusoidal Profile

(A20)
$$z(x)=\frac{h_{c}}{2}\sin\left(\frac{2\pi x}{p}\right)$$

This profile provides smooth curvature transitions and is commonly used in idealized studies.

#### Appendix C.1.2. Semicircular Profile

At the reference *h_c_* = *p*/2, the descending half-period contains two tangent-connected quarter circles. With *a* = *p*/4, *b* = *h_c_*/2 and 0 ≤ *θ* ≤ *π*/2, the first is (*x*, *z*) = (*a *sin *θ*, *b *cos *θ*), and the second is (*x*, *z*) = (2*a* − *a *cos *θ*, −*b *sin *θ*). Their tangent is continuous at *z* = 0, while signed curvature changes. Independent variation of *h_c_* and p turns these arcs into quarter ellipses; the sensitivity data refer to that explicitly scaled family.

#### Appendix C.1.3. Triangular Profile

Piecewise linear function:

(A21) $$z(x)=\left\{\begin{array}{cc} \frac{2h_{c}}{p}x-\frac{h_{c}}{2}, & 0\leq x<\frac{p}{2}\\ -\frac{2h_{c}}{p}x+\frac{3h_{c}}{2}, & \frac{p}{2}\leq x\leq p \end{array}\right.$$

#### Appendix C.1.4. Trapezoidal Profile

Let *α* = 0.50 be the total horizontal-plateau fraction of the period and $a=\alpha \frac{p}{4}$. The descending half follows $\left(0,\frac{h_{c}}{2}\right)$, $\left(a,\frac{h_{c}}{2}\right)$, $\left(\frac{p}{2}-a,-\frac{h_{c}}{2}\right)$, and $\left(\frac{p}{2},-\frac{h_{c}}{2}\right)$. The first and last segments are fully bonded to the rigid faces; only the inclined segment is a free web. The second half is its periodic reflection.

#### Appendix C.1.5. Rounded-Rectangular Profile

Let *r* = 0.10*h_c_*. The descending half starts with a top plateau ending at $\left(\frac{p}{4}-r,\frac{h_{c}}{2}\right)$ and then a clockwise quarter circle centered at $\left(\frac{p}{4}-r,\frac{h_{c}}{2}-r\right)$, a vertical segment at $x=\frac{p}{4}$ ending at $z=-\frac{h_{c}}{2}+r$, a counterclockwise quarter circle centered at $\left(\frac{p}{4}+r,-\frac{h_{c}}{2}+r\right)$, and a bottom plateau ending at $x=\frac{p}{2}$. The arc radii equal r, and the connections are tangent-continuous. This construction requires $0<r<\min\left(\frac{p}{4},\frac{h_{c}}{2}\right)$; its reference $\frac{t_{f}}{r}=0.75$ limits a slender curved-beam interpretation.

#### Appendix C.1.6. Rectangular Profile

The descending half follows $\left(0,\frac{h_{c}}{2}\right)$, $\left(\frac{p}{4},\frac{h_{c}}{2}\right)$, $\left(\frac{p}{4},-\frac{h_{c}}{2}\right)$, and $\left(\frac{p}{2},-\frac{h_{c}}{2}\right)$. Both horizontal segments are fully bonded. The full period contains two free vertical webs of length *h_c_*, with zero end rotation in the *xz* frame model. Its total unfolded length is *L* = *p* + 2*h_c_*.
